# Survey of dermatological conditions in a population of domestic dogs in Mashhad, northeast of Iran (2007-2011)

**Published:** 2013

**Authors:** Javad Khoshnegah, Ahmad Reza Movassaghi, Mehrnaz Rad

**Affiliations:** 1 *Department of Clinical Sciences, Faculty of Veterinary Medicine, Ferdowsi University of Mashhad, Mashhad, Iran; *; 2 * Department of Pathobiology, Faculty of Veterinary Medicine, Ferdowsi University of Mashhad, Mashhad, Iran.*

**Keywords:** Dermatological conditions, Dog; Iran, Prevalence, Survey

## Abstract

university small animal clinic, 1299 Iranian domestic dogs presented from September 2007 through March 2011 to the Ferdowsi University of Mashhad Veterinary Teaching Hospital, were examined. Dermatological disorders were noted in 17.00% (221/1299) of all the dogs examined. Pruritus was the most common presenting sign, accounting for 25.35% of the dermatological consultations. It was followed by erythema, maculo-papular-pustular eruptions (16.97%), erosive or ulcerative lesions (16.74%), scaling or crusting (13.02%), alopecia (8.84%) and visible ectoparasites (7.44%). The most common primary final diagnoses were superficial pyoderma, cutaneous manifestations of canine leishmaniasis, flea infestation and allergy, tick infestation, atopic dermatitis, scabies, unspecified dermatoses, otitis, furunculosis and food allergy. There were no apparent age or sex predilections for dermatological disease as a whole. Spitz (odds ratio = 3.38; *p* = 0.001), Terriers (odds ratio = 2.52; *p *< 0.001) and German Shepherds (odds ratio = 1.90; *p* = 0.001) appeared to be at increased risk for dermatological disease. In addition, Khorasani large cross breed dogs (odds ratio = 0.36; *p* = 0.003) and mixed breed dogs (odds ratio = 0.33; *p* < 0.001) showed decreased risk for dermatological conditions. To the best of our knowledge, this is the first survey study on canine dermatological conditions carried out in Iran.

## Introduction

Several clinical studies have indicated that dermato-logical disorders make up a large proportion of the small animal patients. It has been estimated that from 20.00% to 75.00% of the cases seen in the average small animal practice have skin problems as a chief or concurrent owner complaint.^1^^-^^5^ There is certainly inconsistency among these various studies. For instance, in the methods of describing different types of skin disorders. However, in a recent survey which included the prevalence, diagnosis and treatment of dermatological conditions in small animals in general practice in the UK, a useful practical method for classification of dermatological problem has been explained.^6^ In this survey, out of 3707 small animal consultations in general practice that were observed and recorded, 795 (21.40%) involved animals that had a dermatological problem.

Marked differences were noted in the frequency of the most common skin disorders in the various geo-graphic regions studied.^7^^-^^11^ For example, in a survey of canine and feline skin disorders seen in a university practice in Quebec (Canada), bacterial folliculitis and furunculosis, allergic dermatitis, endocrinopathy, neoplasia, ectoparasitism and immune-mediated dermatitis were found to be the most commonly diagnosed dermatological problems.^3^ However, parasitic infestations, bacterial infections and neoplasia were accounted for the majority of the diagnoses in UK.^6^ Overall, in dogs, flea infestations, bacterial infections, allergic skin diseases, anal sac problems and neoplasia have been reported as the most dermatological conditions.^3^^, ^^7^^-^^11^

To the best of our knowledge, this is the first survey study on canine dermatological conditions carried out in Iran and our objectives were (1) to provide up-to-date information on the prevalence of skin diseases in dogs encountered in a first opinion university small animal clinic and (2) to investigate the most common canine skin disorders in our area. 

## Materials and Methods

**Location of Study. **Mashhad is a metropolitan city locating in northeast of Iran, close to the borders of Afghanistan and Turkmenistan. It is located at 36°20′N 59°35′E in the valley of the Kashaf river, between the two mountains, Binalood and Hezar-Masjed. The city’s climate is semi-arid with cold winters and moderate summers. It has a resident population of approximately 2,500,000.^12^

**Patient selection.** From September 2007 through March 2011, 1299 dogs presented to a first opinion small animal practice (Ferdowsi University of Mashhad, Veterinary Teaching Hospital, Mashhad, Iran), were examined for skin disorders. Six final-year veterinary students who were con-ducting their projects collected data. Three people (including two students and one veterinary dermatologist) were involved in each diagnosis. Seven people were involved over the course of three-and-a-half years. The inclusion criteria for the study were all dogs visited at the clinic and being subject to a dermatological examination as part of their clinical examination, regardless of original presenting complaint. Dermatological cases were defined as any problem that involved the skin, hair or adnexae (nails and claws). Dermatological diagnoses were established by standard criteria.^1^ These included surface sampling (skin scraping, acetate tape impression, flea comb and flotation), hair examination, cytologic examination, examination for fungi (wood’s lamp examination, direct examination and fungal culture), examination for bacteria, biopsy and dermatohistopathologic examination. 

**Dog’s demographic information.** The dogs' breed, sex and age were recorded to determine whether they were associated with the likelihood of dogs exhibiting dermatological problems. The dogs' breed was assessed according to the official breed standard from the American Kennel Club.^13^

**Diagnostic evaluation. **The dermatological signs were classified into one of the following categories as described previously,^6^ with minor modifications: pruritus, alopecia, scaling or crusting, erythema, macular, popular or pustular eruptions, otitis, draining tracts and non-healing wounds, erosive or ulcerative lesions, pigmentary abnormalities, nail disorders, ectoparasites observed by the owner or the clinician, cutaneous swellings and thickening of the foot pads. All masses and swellings involving the skin were classified as dermatological, apart from mammary tumors and swellings that clearly involved other body systems (such as joint effusions or dental abscesses). 

**Diagnostic methods. **Multiple skin scrapings were taken from all the dogs with a history of pruritus. Ectoparasitic infestations were diagnosed by clinical examination, coat brushings, hair plucks and skin scrapings. Other tests included biochemical and hematological profiles, endocrine function tests, impression smears, insect control trials, and skin biopsies, which were also used to diagnose autoimmune skin disorders. Pyoderma and/or *Malassezia* dermatitis were diagnosed using cytology and culture. Cases in which a diagnosis was not made during the consultation were classified as ‘unspecified’. The dermatological cases were further analyzed by investigating the frequency with which different diagnostic tests were undertaken and by determining the prevalence of the different etiological categories and the specific diagnoses. 

A diagnosis or recommendation for treatment was made based on the clinical signs, physical examination and dermatological diagnostic procedures. The most common tests were hematology and biochemistry skin scrapings, otoscopic examination, cytology, bacterial culture and sensitivity, biopsy and coat brushings (Table 1).

**Table 1:** Diagnostic procedures performed to investigate dermatological problems in 181* animals seen in general practice.

**Diagnostic procedure**	**Number**
Hematology/biochemistry	165
Skin scrapings	90
Otoscopic examination	89
Cytology	78
Bacterial culture and sensitivity	62
Biopsy	48
Coat brushings	34
Wood’s lamp	12
Radiography	12
Food trial	11
Total Thyroxine/Free Thyroxine	9
Estrogen/Progesterone	9
Fine-needle aspiration cytology	8
Trichogram	3
**Total**	630

* Except for a small number of patients (40 of 221, 18%), one or more diagnostic procedures were performed in 181 other patients.

**Treatment. **Response to treatment with ivermectin (Razak Pharmaceutical Laboratories, Tehran, Iran) or amitraz (Pfizer Inc., Exton, PA, USA) was used as a part of diagnostic plan. In addition, microbial skin and ear infections were treated with topical and/or systemic antibiotics or antifungal therapy, as appropriate. 

**Data analysis. **Signalments (breed, age, sex) for animals with dermatological diseases were compared with those for the general canine hospital populations during the same time period, and relative risks were calculated as reported previously.^14^ To quantify risk, relative risk was estimated using the odds ratio for the association between selected variables and the outcome. An odds ratio was calculated by dividing the odds (The ratio of the probability of occurrence of an event to that of nonoccurrence) of dermatological diseases in different breeds. Statistical significance was set at *p* < 0.01. All statistical analyses were performed using SPSS 15.0 for Windows (SPSS Inc., Chicago, IL, USA). 

## Results

**Demographic information. **During a three-and-a-half year study period, 221 dogs (with 316 dermatological diagnoses) were diagnosed with one or more skin problems. During the same time period the general hospital population was 1299 dogs. Thus, dermatological disorders accounted for 17.00% of all the dogs examined. One hundred and twenty eight (57.91%) of the dogs were male and 93 (42.09%) were female, with an age and body weight ranging from 3 months to 11.5 years (median: 2.7 years), and from 4.2 to 61.0 kg (median: 21.3 kg), respectively. There were no apparent age or sex predilections for dermatological disease as a whole. One- hundred and eighty-three (82.80%) of these dogs were in 14 different pure breeds. In addition, thirty six (16.28%) dogs were crossbreeds, and two (0.90%) dogs were mongrels. Spitz (odds ratio = 3.38; *p* = 0.001), Terriers (odds ratio = 2.52; *p *< 0.001) and German Shepherds (odds ratio = 1.90; *p* = 0.001) appeared to be at increased risk for dermatological disease. In addition, the following breeds of the dogs appeared to have a decreased risk for dermatological disease: Khorasani large cross breed dogs^-^ (odds ratio = 0.36; *p* = 0.003) and mixed breed dogs (odds ratio = 0.33; *p* < 0.001). 

**Fig. 1:**
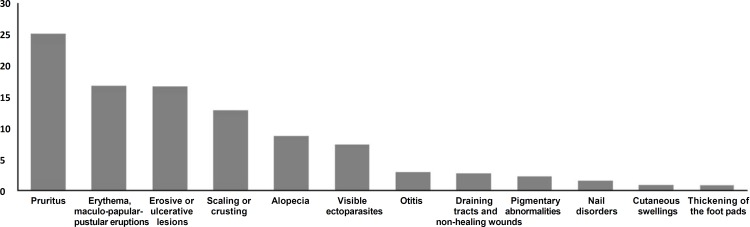
Percentage of the clinical signs in dogs presenting with dermatological problems in the present study.

**Description of dermatological conditions. **The clinical signs in dogs presenting with dermatological problems are shown in Fig. 1. Pruritus was the most common presenting sign, and accounted for 25.35% of all the dermatological consultations, it was followed by erythema, maculo-papular-pustular eruptions (16.97%), erosive or ulcerative lesions (16.74%), scaling or crusting (13.02%), alopecia (8.84%) and visible ectoparasites (7.44%). Other clinical signs such as otitis, draining tracts and non-healing wounds, changes in pigmentation, nail disorders, cutaneous swellings and thickening of the foot pads and the nasal planum were uncommon as primary presentations and accounted for 3.00% or less of the consultations. The specific diagnoses made in all the dermatological cases are shown in Table 2. 

The ten most common primary final diagnoses were superficial bacterial folliculitis (57 of 316, 18.03%), cutaneous manifestations of canine leishmaniasis (36 of 316, 11.39%), Flea infestation/allergy (22 of 316, 6.96%), ticks infestation (18 of 316, 5.69%), atopic dermatitis (16 of 316, 5.37%), scabies (17 of 316, 5.06%), unspecified dermatoses (14 of 316, 4.43%), otitis (13 of 316, 4.11%), furunculosis (13 of 316, 4.11%) and food allergy (10 of 316, 3.16%). 

**Table 2:** Specific dermatological diagnoses made in 221 dogs.

**Diagnosis**	**No.**	**Percentage**	**Diagnosis**	**No.**	**Percentage**
**Bacterial foiliculitis (Superficial) **	57	18.03	**Hyperadrenocortism**	4	1.26
**Cutaneous manifestations of canine leishmaniasis**	36	11.39	**Anal sac impaction/Sacculitis**	3	0.94
**Flea infestation/Allergy**	22	6.96	**Aural hematoma**	3	0.94
**Ticks**	18	5.69	**Demodicosis**	3	0.94
**Atopic dermatitis**	17	5.37	**Hypothyroidism**	3	0.94
**Scabies**	16	5.06	**Miasis**	3	0.94
**Unspecified**	14	4.43	**Acne**	3	0.94
**Otitis**	13	4.11	**Lipoma**	2	0.63
**Furunculosis (Deep)**	13	4.11	**Solar dermatitis**	2	0.63
**Food allergy**	10	3.16	**Interdigital pododermatitis**	2	0.63
***Malassezia*** ** species overgrowth**	7	2.21	**Foreign body**	2	0.63
**Acute moist dermatitis**	7	2.21	**Telogen defluxion**	2	0.63
**Nail infection**	7	2.21	**Psycogenic dermatitis**	2	0.63
**Alopecia X**	7	2.21	**Trichoblastoma**	1	0.31
**Primary Seborrhea**	6	1.89	**Wart**	1	0.31
**Abscess**	5	1.89	**Injection site reaction**	1	0.31
**Acral lick dermatitis**	5	1.89	**Dermatophytosis**	1	0.31
**Contact allergy**	5	1.89	**Fly dermatitis**	1	0.31
**Lice** ** (Pedicolosis)**	5	1.89	**Drug eruption**	1	0.31
**Cyclic flank alopecia**	5	1.89	**Erythema multiforma**	1	0.31
			**Total** *	316	100

* Total number of dogs is greater than 221, because some of them had more than one condition.

## Discussion

The present survey study provides an insight into the prevalence and clinical aspects of dermatological conditions in a population of Iranian domestic dogs in and around the city of Mashhad (northeast of Iran) and some of the factors that may be associated with the occurrence of dermatological problems in a veterinary clinical sample of domestic dogs.

The results of the present study indicated that the dermatological cases accounted for approximately 17.00% of the small animal consultations seen during a three-and-a-half year study survey in a first opinion small animal practice. In other studies, the prevalence of canine skin conditions was reported as being between 15.00% and 25.00%.^2^^,^^3^^,^^6^ This similarity suggests despite considerable improvements in the provision or prophylactic health care, skin conditions requiring veterinary intervention are still as common as they were in the 1970s. 

The main dermatological problems in dogs encountered in the present study were superficial bacterial folliculitis, flea infestation/allergy, ticks infestation, atopic dermatitis, scabies, unspecified dermatoses, otitis, furunculosis and food allergy. In some cases, these differ from those identified by other studies. Scott and Paradis^3^ found that bacterial folliculitis and furunculosis, allergic dermatitis, endocrinopathy, neoplasia, ectoparasitism and immune-mediated dermatitis were the most commonly diagnosed dermatological problems. Also, according to Hill *et al.* parasitic infestations, bacterial infections and neoplasia accounted for the majority of the diagnoses.^6^

In the current study, we have also examined the frequency with which different presenting signs were observed. This ‘problem-oriented approach’ can be found in a number of recent dermatological texts.^15^^-^^17^ In addition, it relates the way the animal presents in the consulting room to the possible differential diagnoses, something that is not possible with an aetiological classification. Using a modified version of Hill *et al.* method, the presenting clinical signs in descending order of frequency were pruritus, maculo-papular-pustular eruptions, erosive or ulcerative lesions, scaling or crusting, alopecia, visible ectoparasites, otitis, draining tracts and non-healing wounds, changes in pigmentation, nail disorders and cutaneous swellings.^6^ Parasitic infestations and allergies were the predominant causes of the two main presenting signs in our study, pruritus and maculo-papular-pustular eruptions. The specific diagnoses that were made most commonly in animals with pruritus were flea infestation, atopic dermatitis, acral lick dermatitis, *Malassezia *species dermatitis and acute moist dermatitis. In the animals with erythema, maculo-papular-pustular eruptions, different kind of allergies and leishmaniasis were most commonly diagnosed. Most of these conditions figure highly in previous studies of the prevalence of skin diseases in dogs.^3^^,^^7^^-^^11^^,^^18^

The laboratory investigation of the dermatological problems was limited in the cases reported by Hill *et al.*, in which 72.00% of the cases diagnosed with no diagnostic evaluation except for a physical examination.^6^ Although many skin problems can be diagnosed on the basis of clinical signs alone, this figure seems high. The lack of diagnostic testing may have accounted for the fact that in over 20.00% of the dogs in the mentioned study, no specific etiological diagnosis was made, and it may also have resulted in some cases being misdiagnosed. In the present study, 18.00% of the cases were diagnosed in this way (no diagnostic evaluation except for a physical examination). In a large number of studied dogs, we used invaluable methods such microscopic examination of skin scrapings, cytological specimens, fine-needle aspirates and biopsies. These techniques should be used more frequently in dermato-logical investigations by new veterinary graduates and more experienced practitioners alike. 

The results of this study provided valuable data on the prevalence, investigation and treatment of skin disorders in general practice. The data were collected from a university teaching hospital in Iran, and should not therefore have been biased by major national variations, and over a period of three-and-a-half years covering all four seasons, so that seasonal variations should have been covered.
